# Stacking and energetic contribution of aromatic islands at the binding interface of antibody proteins 

**DOI:** 10.1186/1745-7580-6-S1-S1

**Published:** 2010-09-27

**Authors:** Di Wu, Jing Sun, Tianlei Xu, Shuning Wang, Guoqing Li, Yixue Li, Zhiwei Cao

**Affiliations:** 1 Department of Biomedical Engineering, College Life Science and Technology, Tongji University, Shanghai, 200092, China; 2 Shanghai Center for Bioinformation Technology, Qinzhou Rd 100, Building 1, 12F, Shanghai, 200235, China; 3 Bioinformatics Center, Key Lab of Systems Biology, Shanghai Institutes for Biological Sciences, Chinese Academy of Sciences, Shanghai 200031, China

## Abstract

**Background:**

The enrichment and importance of some aromatic residues, such as Tyr and Trp, have been widely noticed at the binding interfaces of antibodies from many experimental and statistical results, some of which were even identified as “hot spots” contributing significantly greater to the binding affinity than other amino acids. However, how these aromatic residues influence the immune binding still deserves further investigation. A large-scale examination was done regarding the local spatial environment around the interfacial Tyr or Trp residues. Energetic contribution of these Tyr and Trp residues to the binding affinity was then studied regarding 82 representative antibody interfaces covering 509 immune complexes from the PDB database and IMGT/3Dstructure-DB.

**Results:**

The connectivity analysis of interfacial residues showed that Tyr and Trp tended to cluster into the spatial Aromatic Islands (AI) rather than being distributed randomly at the antibody interfaces. Out of 82 antibody-antigen complexes, 72% (59) interfaces were found to contain AI with more than 3 aromatic residues. The statistical test against an empirical distribution indicated that the existence of AI was significant in about 60% representative antibody interfaces. Secondly, the loss of solvent accessible surface area (SASA) for side chains of aromatic residues between actually crowded state and independent state was nicely correlated with the AI size increasing in a linearly positive way which indicated that the aromatic side chains in AI tended to take a compact and ordered stacking conformation at the interfaces. Interestingly, the SASA loss of AI was also correlated roughly with the averaged gap of binding free energy between the theoretical and experimental data for immune complexes.

**Conclusions:**

The results of our study revealed the wide existence and statistical significance of “Aromatic Island” (AI) composed of the spatially clustered Tyr and Trp residues at the antibody interfaces. The regular arrangement and stacking of aromatic side chains in AI could probably produce extra cooperative effects to the binding affinity which was firstly observed through the large-scale data analysis. The finding in this work not only provides insights into the functional role of aromatic residues in the antibody-antigen interaction, but also may facilitate the antibody engineering and potential clinical applications.

## Background

It is well known that protein-protein interactions are fundamental to most of biological processes, including signal transduction, gene translation or transcription, enzyme activation or inhibition, and immune recognition. Contrast to the interaction between other normal protein-protein complexes, the binding between antibody and antigen is highly specific and stable [1]. Previous studies have revealed that this specificity is dominantly determined by the contacting interface which is mainly composed of the variable domains of antibody [2-6]. It has been reported that with only 5% sequence change in the variable domains, antibodies can recognize specifically and bind tightly to 10^10^ different antigens [7]. It is always interesting to study how antibody can recognize so large variety of antigens with so little change in sequence and thus deserve further investigation. 

Characteristics of the binding interfaces of antibodies such as the size, shape, chemical, physical or structural complementation have been analyzed from different perspectives for a deeper understanding to antibody-antigen interactions [8-10]. Although the hydrophobic effect was considered as the major driving force for the general protein binding, the study of Tsai and co-workers indicated that hydrophobic amino acids were not the dominant part and a higher proportion of charged and polar residues could be found at the binding interfaces [11]. Subsequent comparison between the interfaces of six antibody-antigen complexes and other protein-protein complexes reported that the residues composing the interface of antibody-antigen complexes were more polar, protruding and accessible [12]. Currently, more and more results suggest that there are significant differences between the interfaces of immune and non-immune protein complexes. For instance, the interfaces of antigen-antibody complexes are particularly rich in Tyr, Arg, His, Phe and Trp [13-17]. Although further observations indicate that this enrichment ranking alters slightly with different data size, aromatic residues have always been found to occur more frequently at the binding sites of antibodies. 

On the other hand, the contribution of enriched residues to the binding selectivity and specificity of antibody has aroused extensive interest [18]. By the virtue of alanine scanning mutagenesis, the energetic contribution of respective residue to protein binding could be evaluated with the observed free energy variation derived from the introduced mutation [19-22]. The results of mutations have frequently indicated that the affinity change of mutating certain interfacial residue is far more unpredictable which is considered as the hot spot residue at the binding interface [23]. Some interfacial Tyr or Trp residues, but not all of them, have subsequently been identified as hot spots that contribute significantly to the high affinity of antibody-antigen interactions [24]. Despite that the different conclusions have been derived from several individual experiments adopting different datasets and methodologies, the enrichment and important role of Tyr and Trp residues have been widely noticed at the binding interfaces of antibodies. 

However, several questions are still open to be answered. Why are these aromatic residues enriched and preferred? How do they affect the affinity so largely and form the “hot spots”? Are there any special local environment existing around the Tyr or Trp residues to facilitate the interaction at the interface? … In order to answer these questions, an in-depth and large-scale analysis would be helpful focusing on the aromatic residues at the binding interfaces of antibody-antigen complexes. Here, we conducted a comprehensive analysis of 82 non-redundant interfaces of antibodies covering 509 immune complexes from the PDB database [25] and IMGT/3Dstructure-DB [26,27]. The residue connection and spatial distribution were scanned for all interfacial Tyr and Trp residues following an enrichment analysis. Systematic study was further focused on the relationship between the distribution pattern and the energetic contribution of aromatic interfacial residues in order to reveal the function of the aromatic residues in the binding interfaces of antibodies. 

## Results

### “Aromatic Island” at the interface of antibodies

As to the antibody-antigen complexes collected in our dataset, the area of binding interfaces was firstly calculated. In general, the loss of solvent accessible surface area (SASA) was about 173~2351Å^2^ for antibodies upon the complex formation, and the average value was 644 Å^2^. The residue composition for interface of antibodies was also calculated. According to the absolute value of residue composition, Tyr, Ser, Thr were the top 3 most abundant residues at the interfacial area of antibodies with 17.10%, 14.61% and 8.66% respectively (Additional file 1). Removing the intrinsic abundance of residues in whole antibody, Tyr, Arg, and Trp were found to be the top 3 significantly enriched amino acids at the binding interface of antibodies (Additional file 1). Our finding is consistent with the previous results, especially for the significant enrichment of Tyr and Trp residues at the binding interface of antibodies [14,15]. Different from Trp, Tyr residues are not only significantly enriched and preferred at the antibody interface, but also highly abundant for residue composition of antibody interface in the absolute value.

**Figure 1:**
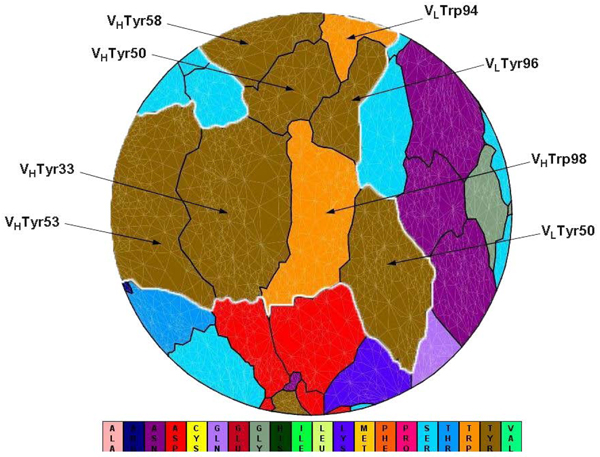
**Flatten view of a binding interface at an antibody (PDB code 1c08).** V_L_Tyr50, V_L_Trp94, V_L_Tyr96, V_H_Tyr33, V_H_Tyr50, V_H_Tyr53, V_H_Tyr58, V_H_Trp98 (IMGT numbering: V-Kappa Tyr56, V-Kappa Trp114, V-Kappa Tyr116, VH Tyr38, VH Tyr55, VH Tyr58, VH Tyr66, VH Trp107) cluster together to form a spatial “Aromatic Island” (abbreviated to “AI”) at the HyHEL-10 antibody interfaces, which is highlighted with white line (Tyr, colored in dark yellow; Trp, colored in orange). Figure 1 is generated by software JMiV.

In the following, the local environment was studied through residue connectivity study around all Tyr and Trp interfacial residues at the 82 antibody interfaces. Surprisingly, careful review of the results indicated that Tyr and Trp residues tended to cluster together to form a spatial “Aromatic Island” (abbreviated to “AI”) at the antibody interfaces, rather than being scattered or distributed randomly among the interface. A typical example can be found in Figure 1. Out of 82 antibody interfaces, 72% (59 interfaces) were found to contain AI with size above 3 aromatic Tyr or Trp residues (Additional file 2). The biggest cluster contained 12 aromatic residues in the antibody binding site, such as the case in complex with PDB code 1bgx. In general, among all Tyr and Trp interfacial residues at the binding interfaces of 82 antigen-antibody complexes, there are about 76% residues clustering into the AI. By the way, tracing back to 509 antibody interfaces, 68% (347) interfaces were found to contain AI with size above 3 Tyr or Trp residues. 

### Statistical analysis of “Aromatic Island”

From the previous residue composition of antibody interface, the aromatic residues of Tyr and Trp are dominant which means there could be a large number of Tyr and Trp residues at the binding interface. Under such circumstance, the occurring probability for observing the connecting aromatic residues is increasing at the interface. Thus, it’s necessary to estimate whether the AI phenomenon is of statistical significance or simply the result of Tyr or Trp residues enrichment at the binding interface. Excluding the 17 interfaces with no clustered Tyr and Trp residues, the remaining 65 interfaces were simulated. After 10,000 times of simulation, the distribution pattern of re-arranged aromatic interfacial residues at the simulative interfaces were investigated. For every interface, the statistical test against an empirical distribution was made, and the likelihood was calculated among 10,000 simulations for observing the AI with the same residue composition and size as the actual interface. The results were summarized in Table 1 (the detail probability for every interface were recorded in Additional file 2). 

**Table 1:** Statistical Probability for Aromatic Island among simulative interfaces

Size of AI^a^	Number of Antibody Interfaces	Averaged Probability^b^	Number of Interfaces with statistically significant AI	Averaged Percentage^c^
2	7	51.01%	0	40.0%
3	6	23.93%	0	51.4%
4	15	3.45%	12	75.0%
5	9	1.68%	9	81.8%
6	11	0.21%	11	94.3%
7	9	0.08%	9	94.0%
>= 8	8	0	8	94.6%
Total	65	/	49	/
				

^a^ The size of AI is indicated in terms of the number of aromatic Tyr and Trp residues in AI

^b^ The averaged probability for observing the AI among simulative interfaces (% for 10,000 times)

^c^ The averaged percentage for interfacial aromatic residues being clustered into AI

Generally, the probability to observe AI among simulative interfaces dropped sharply from 51% to 0 with the AI size increasing from 2 to 12 residues. When the AI size is below 4 aromatic residues in actual interfaces, the probability for observing such AI was not statistically significant (the probability > 5.0%, statistical test against an empirical distribution). Furthermore, it was found that the statistical significance to observe AI was not only related to the AI size, but also related to the percentage of aromatic interfacial residues being clustered into AI. For example, there were 3 interfaces whose probabilities for observing AI were not statistically significant among 15 interfaces with AI size of 4 aromatic residues (Table 1). One reason could be that abundant aromatic residues were observed at the interface, but only less than 70% were included into AI.  

Among all the 65 interfaces, 49 interfaces (75.4%) were found with significant AI when compared with the simulative interfaces (the probability < 5.0%, statistical test against an empirical distribution). In summary, the significant AI was observed at 60% of representative antibody interfaces (49/82). Thus, the majority of AI at the binding interfaces of antibodies were of statistical significance. 

### Regular arrangement of aromatic side chains in “Aromatic Island”

As we know that, both Tyr and Trp residues have bulky and rigid ring chain structure, comparing to most of other amino acids with either small or flexible side chains. According to previous research, the extending conformation would be preferred for an aromatic residue in open surrounding [28,29]. How are the side chains arranged when so many aromatic rings crowd together in the AI at the binding interface of antibody? Since the compactness of side chains for aromatic residues in AI could be reflected by the loss between the sum of SASA for every side chain of aromatic residue in fully independent state and the actual SASA for AI in crowded state, we calculated the loss of SASA for every AI observed in all antibody interfaces. If the aromatic rings keep some compact and regular arrangement in AI, the loss of SASA would be correlated with AI size. Contrarily, the random arrangement of aromatic rings will not result in a nice correlation.  

**Figure 2:**
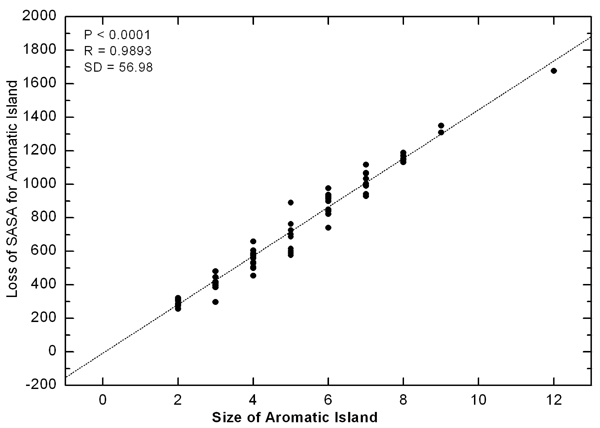
**Correlation between the size of Aromatic Island and the loss of SASA for Aromatic Island** The loss of SASA for AI (Å^2^) is calculated as the difference between the sum of SASA for every side chain of aromatic residue in fully independent state and the actual SASA for Aromatic Island. The size of Aromatic Island is recorded as the number of aromatic Tyr and Trp residues included in AI

**Figure 3:**
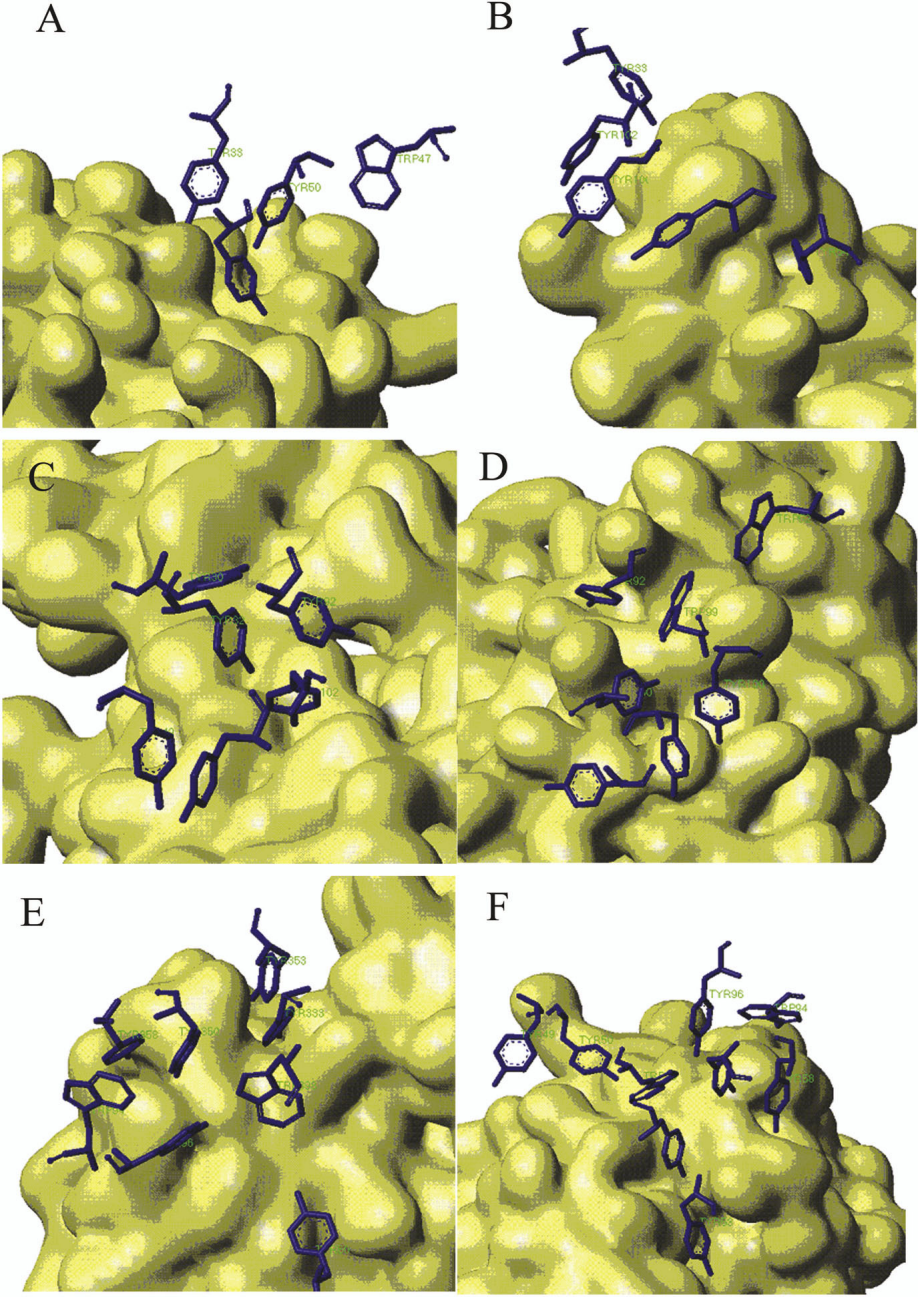
**Stacking Conformation for aromatic residues in Aromatic Island** The antigen protein is displayed with solvent surface mode. For better view, only the aromatic interfacial Tyr and Trp residues in AI are displayed with stick mode from the antibody. (A) Aromatic Island size of 4 at the binding interface of antibody 9D7 and IL-10 (PDB code 1lk3) (B) Aromatic Island size of 5 at the binding interface of antibody 33H1 and potassium channel molecule (PDB code 1ors) (C) Aromatic Island size of 6 at the binding interface of antibody and cytochrome AA3 (PDB code 1ar1) (D) Aromatic Island size of 7 at the binding interface of antibody YTS 105.18 and T-cell surface glycoprotein CD8 alpha chain (PDB code 2arj) (E) Aromatic Island size of 8 at the binding interface of antibody HyHEL-26 and HEL (PDB code 1ndm) (F) Aromatic Island size of 9 at the binding interface of antibody HyHEL-10 mutant and HEL (PDB code 2eiz)

The sum of SASA for every aromatic residue in independent state was calculated by multiplying the number of Tyr and Trp residues included in AI and the corresponding SASA for the side chain of extending Tyr or Trp residue, which is determined as 182.8 Å^2^ or 208.8 Å^2^ for Tyr or Trp in tri-peptide ala-X-ala respectively [30]. The loss of SASA was plotted to AI size in Figure 2. As can be seen from the chart, the deviation was positively correlated with the AI size increasing in a linear way, which suggested that the aromatic rings tend to keep compact and regular arrangement in AI. In order to check what the regular pattern could be for aromatic side chains, we re-inspected the surface structures of aromatic residues in AI at the binding interfaces of all antibodies, and the representative results were shown in Figure 3. From Figure 3A, 3B, 3C, 3D, 3E, 3F, it was noticed that the aromatic side chains in AI tended to stack or pile the aromatic rings together and extended their trends along the cleft on the binding sites of antigen interface. The more typical pattern could be seen in Figure 3E and Figure 3F.

### Energetic contribution of “Aromatic Island” to binding affinity

It is well known that the abundant stacking of aromatic rings can produce strong and extra effects to stabilize the structures for nucleic acids [31,32]. The regular stacking of aromatic side chains at antibody interface reminds us to verify whether such cooperative effect also exists during the binding of antibody and antigen. 

Normally, it is realized that the experimental mutational data is the final effect observed. However, the computed data is actually a theoretical sum-up of individual mutation, where the cooperative effects between neighboring residues cannot be completely integrated. The experimental binding free energy for antibody-antigen complex is the result of global effect which covers the contribution of AI, while the computationally obtained binding free energy is usually the summary of individual residue effect. Under the same systemic error, the gap of binding free energy between the experimental and computational results might to some extent reflect the collective effect of AI if the stacking of aromatic rings does contribute to antibody-antigen binding.  

**Figure 4:**
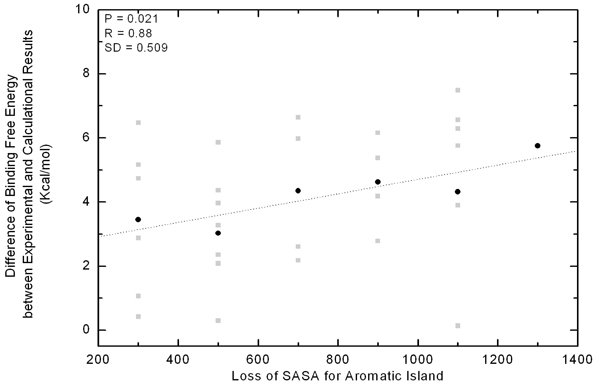
**Correlation between the energetic gap and SASA loss of Aromatic Islands at 30 antibody interfaces.** Along the x-axis all of the values in a bin (size 200 Å^2^) are pulled together as a group and shown in the middle. The gap of binding free energy between theoretical and experimental data is indicated with grey square for every immune complex. In each group, the gap is averaged and indicated with black dot.

After the comprehensive literature review, the experimental binding free energy of 30 immune complexes was obtained (Additional file 3). The absolute value of energy gaps between experiment and theoretical calculation was plotted according to the compactness of AI, as being illustrated by dot in Figure 4. The compactness of AI was again estimated by the loss of SASA sum for aromatic residues in AI between the independent state and the actual interface. The theoretical binding free energy was calculated for all the 30 immune complexes through the same procedure as being described in method part. It can be seen from Figure 4, the average value of energy gap between the theoretical and experimental data roughly correlated with the AI compactness increasing in a linear way. 

## Discussion

In this paper, the spatial distribution of enriched aromatic Tyr and Trp residues was investigated where compact and significant AI was widely found at most of the binding interfaces of antibodies. In previous experiments, several cases have been reported that Tyr or Trp plays an important role in various aspects, such as increasing the affinity and helping induce fit of antibody to antigen proteins [18-21]. However, the utilization of individual residues seems not enough to fully explain the prevalence and enrichment of Tyr and Trp. The existence of AI at the antibody interfaces is acceptable to explain both above. Firstly, Tyr and Trp have been found to be able to form more close interactions to antigen proteins than other peers because of the aromatic and hydroxyl groups in their side chain [21,28]. Interestingly, Phe is another amino acid with aromatic side chain, and was rarely found at the antibody interface. It is possibly because the absence of functional group in the aromatic ring.

Furthermore, the piling of aromatic rings may produce extra stabilizing energy for the whole structures of protein complexes, similar to the case in nucleic acids. In addition, the dense gathering of aromatic side chains could cause a highly hydrophobic local environment where hydrophobic interaction was found to contribute most to the antigen-antibody binding probably because of the exclusion of solvent from nearby polar interactions [22].

It can be seen from Figure 4 that more aromatic residues are found in AI, more energetic deviation can be observed between the computational and experimental results under the same systemic error. If the aromatic interfacial residues in AI function separately without the cooperative effects between each other, the energetic contribution of them will be roughly additive so that the positive and linear correlation will not be observed. In summary, the rough correlation in Figure 4 suggested that there may be some unknown cooperative effects existing within the AI and these effects will increase with more and more Tyr or Trp residues stacking together. In other words, if one aromatic residue in AI is mutated into non-aromatic, the introduced effects to the binding affinity could be much larger than our expectation. This finding has gained supports from many instances [33-35]. For example, the mutation of an aromatic residue in AI of V_L_Trp92 (IMGT number: V-KAPPA Trp108 [36]) to Leu could lead to 1,000 fold decreases in binding affinity in the complex of D1.3/HEL [33]. In another interesting study of two antibodies to the antigen vascular endothelial growth factor (PDB code 1cz8 and 1bj1), one single mutation of V_H_His101 (IMGT number: VH His109) to Tyr would cause 14 fold of affinity enhancement [34,35]. Our further analysis found this mutation will cause the AI size expanding from 4 to 7. 

As to the computational methodologies to calculate the binding free energy, there have been several methods available such as MM/PBSA and other methods [37]. Although molecular dynamics methodology may give more accurate calculation [38-41], these methods are avoided considering that the computing time is overwhelming for 82 antibody-antigen complexes. No matter what method is adopted, the trend for deviation between the computational and experimental results has certain reliability under the same systemic error. Similar trend was also obtained by an alternative method Rosetta (Additional file 4 - Figure S1) [42]. 

## Conclusion

In summary, a large-scale analysis is done focusing on the spatial distribution and energetic contribution of aromatic interfacial residues for representative antibody interfaces. The results of this paper reveal the wide existence of “Aromatic Island” at the antibody interfaces where Tyr and Trp residues cluster together in an ordered way along the cleft of antigen surface. The gathering of aromatic side chains probably produces an extra cooperative effect which contributes significantly to the binding affinity between antigen and antibody. The finding of “Aromatic Island” to some extent supports the “molecular crowding” speculation in the association interface. On the other hand, the collective effects resulted from the aromatic side chains might also form the optimized local environment, which could flexibly accommodate and recognize a variety of spatial epitopes, and stabilize the molecular complexes. Future work on their exact function will not only benefit the intricate mechanism of immune recognition and specificity, but also facilitate the antibody engineering, antigen docking and potential clinical applications.

## Methods

### Dataset of antigen-antibody complexes

Four hundred and sixty four structures of antibody and protein antigen complexes have been obtained from the Protein Data Bank, dated Aug, 2008 and IMGT/3Dstructure-DB. In Jan, 2010, the PDB database was rescanned. 45 new crystal structures of antibody-antigen complexes were deposited and appended in our dataset. Totally, there were 509 antibody-antigen complexes included in our dataset. Those with resolution better than 3.0Å and length of antigen molecules more than 25 residues were requested to guarantee the protein antigen instead of peptide antigen. Considering that antibodies have highly similar sequence similarity, the redundancy is removed according to the spatial epitopes of protein antigen. In our study, strict criteria were set to compare the similarity of spatial epitopes between different complexes. If the identical interfacial residues between two spatial epitopes are above 20%, only one complex with the best resolution is kept. The final dataset of immune complexes was composed of 82 antibody-antigen complexes. The detailed list can be found in Additional file 2 for the PDB IDs of antibody-antigen complexes included in our dataset. 

Within these complexes, the interfacial residues of antibody were defined according to the change of solvent accessible surface area (Δ*SASA*) for each residue in unbound and bound structures [43]. 

Δ*SASA* = *SASA_unbound_ – SASA_bound_*                  (1)

The value of solvent accessible surface area was calculated with program NACCESS [44]. The radius of water probe was set to be 1.4 Å [15]. The unbound structure of proteins derived directly from the structure file of complexes. The interfacial residues were those with Δ*SASA* more than 1Å^2^.

### Residue connectivity at the antibody interface

The computational alanine scanning mutagenesis method was adopted to retrieve the residue contacts for the antibody interface. The interfacial residues of antibodies were mutated into Ala one by one. Comparing the SASA for every interfacial residue between mutants and wild type, the feature of spatial contact between interfacial residues of antibody was determined according to the change of SASA. Through the spatial contiguity, the distribution pattern for interfacial residues at the antibody interface was analyzed.

### Random simulation of residue re-arrangement for antibody interface

To study whether the distribution pattern for clustered aromatic residues is of statistical significance, especially when the number of aromatic residues increases at the interface of antibody, the random re-arrangement of interfacial residues was made for simulative interface and compared to the actual interface of antibody. With the same solvent accessible surface area and residue composition, the interfacial residues and whole binding interface were simplified as the circles. The substituted circle for every residue was rigid, and the radius for every circle could be determined with the formula to calculate the area of circle, *S = π×r^2^*, where *S* was the area of substituted circle and equal to the SASA, and *r* was the radius. Then, the computational simulation was processed to simulate the pattern of random re-arrangement for interfacial residues. The substituted circles for interfacial residues were randomly laid in the big circle representing the whole interface of antibody. During the simulation, the first one circle was laid at the center. Then, the following circles were randomly laid nearest to the center one. Until all circles were laid into the big circle, a complete simulation was finished. Considering the gaps between the individual artificial circles, the boundary of the big circle is set to be flexible and exceeding of 5 Å is allowed. Every binding interface of antibody was simulated 10,000 times. The distribution of aromatic Tyr and Trp interfacial residues were investigated with comparison between the actual interfaces and the simulated ones. The statistical significance of the AI phenomenon was inspected with a test against the empirical distribution among the simulated binding interfaces. The likelihood was directly calculated among 10,000 simulations for observing the AI with the same residue composition and size as the actual interface When the probability was less than 5.0%, the AI phenomenon at the interface of antibody is considered with the statistical significance.

### Calculation of binding affinity for immune complexes

In order to discuss the possible energetic contribution derived from the enriched aromatic interfacial residues, molecular modeling methods were adopted to calculate the binding affinity for immune complexes. The binding free energy was selected to evaluate the binding affinity in our research. The 3-D structure of complexes from PDB was prepared by adding hydrogen atoms in InsightII program package. Then structure was optimized by 500 steps of steepest decent followed by 1000 steps of adopted basis Newton Raphson under CHARMM force field. In the minimization process, explicit solvent molecules TIP3 were used to solvate the antibody-antigen complex. Optimized structure was saved for subsequent calculation of the binding affinity, while the water molecules were discarded. Program of DCOMPLEX was used to calculate the binding free energy [45]. On the other hand, the experimental binding affinity was derived based on the binding constant in literature, including *k_on_*, *k_off_*, *K_D_* and *K_A_*, calculated following the thermodynamic method:

*ΔG = -RTln K_A_*                          (4)

*K_A_ = 1/ K_D_ = k_on _/ k_off_*                       (5)

## Competing interests

The authors declare that they have no competing interests

## Authors’ contributions

DW carried out the program design, performed the statistical analysis and drafted the manuscript. JS carried out the data collection and the statistical analysis. TLX, SNW and GQL performed the statistical analysis. YXL helped to draft the manuscript. ZWC drafted the manuscript. All authors read the final manuscript 

## Supplementary Material

Additional file 1 Table S1 Residue Composition for the interface of antibody Click here for file

Additional file 2 Table S2 Aromatic Island in Binding Interfaces of Antibodies and Statistical Probability among simulative interfaces Click here for file

Additional File 3 Table S3. Experimental Binding affinity for immune complexes Click here for file

Additional file 4 Figure S1 Correlation between energetic gap and SASA loss of Aromatic Islands at 30 antibody interfaces. Click here for file
