# Supplementary material for: Stacking and energetic contribution of aromatic islands at the binding interface of antibody proteins
Source: Immunome Res. 2010 Sep 27;6(Suppl 1):S1. doi: 10.1186/1745-7580-6-S1-S1 (PMC2946779; doi:10.1186/1745-7580-6-S1-S1)
Supplement: Additional file 1 — Table S1 Residue Composition for the interface of antibody [file 1745-7580-6-S1-S1-S1.pdf]

Additional file 1

Table S1 Residue Composition for the interface of antibody

Residue Type	Residue Composition		
	Absolute Composition (%)		Relative Propensity
	Interface	Whole Antibody	
Ala	2.12	6.052	0.35
Arg	4.92	2.889	1.70
Asn	8.30	3.610	2.30
Asp	7.35	4.285	1.72
Cys	0.05	2.053	0.02
Gln	2.21	4.532	0.49
Glu	3.61	3.744	0.96
Gly	7.31	7.491	0.98
His	2.62	1.417	1.85
Ile	1.85	3.257	0.57
Leu	3.25	6.861	0.47
Lys	3.52	5.392	0.65
Met	0.45	1.144	0.39
Phe	2.57	3.239	0.79
Pro	1.99	5.514	0.36
Ser	14.61	14.355	1.02
Thr	8.66	9.745	0.89
Trp	5.14	2.120	2.43
Tyr	17.10	4.976	3.44
Val	2.39	7.324	0.33
