# Supplementary material for: Stacking and energetic contribution of aromatic islands at the binding interface of antibody proteins
Source: Immunome Res. 2010 Sep 27;6(Suppl 1):S1. doi: 10.1186/1745-7580-6-S1-S1 (PMC2946779; doi:10.1186/1745-7580-6-S1-S1)
Supplement: Additional file 2 — Table S2 Aromatic Island in Binding Interfaces of Antibodies and Statistical Probability among simulative interfaces [file 1745-7580-6-S1-S1-S2.pdf]

Additional File 2

Table S2 Aromatic Island in Binding Interfaces of Antibodies and Statistical Probability among simulative interfaces

No.	PDB ID	Number of Tyr/Trp residues in interface	Aromatic Interfacial Residues in AI	Probability for Statistically significant AI among simulative interfaces
1.	1a14	5	V_LTyr32, V_LTyr50, V_HTyr52, V_HTyr99	3.87%
2.	1a2y	7	V_LTyr32, V_LTyr49, V_LTyr50, V_LTrp92, V_HTrp52, V_HTyr 101	0.65%
3.	1ahw	6	V_LTyr32, V_LTyr50, V_HTyr32, V_HTyr33, V_HTyr102	0.24%
4.	1ar1	7	V_LTyr30, V_LTyr32, V_LTyr49, V_LTyr92, V_HTyr101, V_HTyr102	0.62%
5.	1bgx	16	V_LTyr32, V_LTyr34, V_LTyr36, V_LTrp91, V_LTyr94, V_HTrp47, V_HTyr50, V_HTyr96, V_HTyr97, V_HTyr99, V_HTrp100, V_HTyr101	0.00%
6.	1bj1	8	V_LTrp96, V_HTyr32, V_HTrp50, V_HTyr99, V_HTyr102, V_HTyr103, V_HTrp108	0.04%
7.	1dqj	8	V_LTyr50, V_LTrp94, V_LTyr96, V_HTyr33, V_HTyr50, V_HTyr53, V_HTyr58, V_HTrp98	0.00%
8.	1dzb	7	V_LTyr33, V_LTrp99, V_LTrp101, V_LTyr102, V_LTyr232, V_LTyr250, V_LTyr296	0.05%

9.	1e6j	6	V_LTyr92, V_HTyr50, V_HTyr105	28.09%
10.	1egj	4	/	/
11.	1eo8	5	V_LTyr49, V_HTrp100	62.79%
12.	1fbi	7	V_LTyr32, V_LTyr92, V_HTyr32, V_HTrp33, V_HTyr57	3.47%
13.	1fe8	7	V_LTyr32, V_LTyr49, V_LTyr50, V_LTrp96, V_HTrp52, V_HTyr99, V_HTyr100	0.02%
14.	1fj1	5	V_LTyr32, V_LTyr50, V_LTyr91	22.87%
15.	1fns	6	V_LTyr32, V_LTrp96, V_HTrp266, V_HTyr316, V_HTyr319, V_HTyr321	0.08%
16.	1g9m	2	/	/
17.	1g9n	2	/	/
18.	1h0d	4	V_LTyr30B, V_HTyr98, V_HTyr100B	11.32%
19.	1igc	1	/	/
20.	1iqd	4	V_LTyr33, V_LTyr92	59.08%
21.	1jhl	8	V_LTyr49, V_LTyr94, V_LTrp96, V_HTyr32, V_HTrp33, V_HTyr52, V_HTyr57, V_HTyr102	0.00%
22.	1jps	5	V_LTyr32, V_LTyr50, V_HTyr32, V_HTyr33	4.57%
23.	1jrh	8	V_LTyr30, V_LTrp92, V_LTrp96, V_HTyr32, V_HTrp52, V_HTrp53, V_HTyr58	0.07%

24.	1kb5	6	V_LTyr32, V_LTyr92	45.66%
25.	1lk3	5	V_HTyr33, V_HTrp47, V_HTyr50, V_HTyr57	3.37%
26.	1mhh	0	/	/
27.	1mhp	4	V_LTrp90, V_LTrp95, V_HTrp47, V_HTyr58	0.48%
28.	1mlc	5	V_LTrp94, V_HTyr32, V_HTrp33, V_HTyr59	3.35%
29.	1nl0	3	V_LTrp91, V_HTyr58	/
30.	1n8z	7	V_HTyr33, V_HTyr52, V_HTyr57, V_HTrp99, V_HTyr105	1.61%
31.	1nca	6	V_LTyr49, V_LTrp50	64.20%
32.	1ndg	7	V_LTyr50, V_LTrp94, V_LTyr96, V_HTyr333, V_HTyr350, V_HTrp398	0.05%
33.	1ndm	8	V_LTyr50, V_LTrp94, V_LTyr96, V_HTyr333, V_HTyr350, V_HTyr353, V_HTyr358, V_HTrp398	0.00%
34.	1nfd	4	V_HTrp33, V_HTyr35	45.16%
35.	1nsn	7	V_LTyr32, V_LTyr50, V_LTyr96, V_HTyr50	5.94%
36.	1oaz	9	V_LTyr34, V_HTyr32, V_HTrp33, V_HTrp100, V_HTyr101, V_HTyr102, V_HTyr105, V_HTyr106	0.00%
37.	1ob1	5	V_LTrp50, V_LTyr96, V_LTyr97, V_HTyr96	2.90%
38.	1ors	6	V_LTyr33, V_HTyr33, V_HTyr54, V_HTyr100, V_HTyr102	2.16%

39.	1osp	6	V_LTyr30, V_LTrp92, V_HTyr101, V_HTyr102	1.94%
40.	1ots	7	V_LTrp90, V_HTrp33, V_HTyr100, V_HTyr101, V_HTyr103, V_HTyr105, V_HTrp106	0.01%
41.	1pkq	3	/	/
42.	1qfu	5	/	/
43.	1qfw	6	V_LTyr100, V_LTyr102, V_HTyr32, V_HTrp33, V_HTyr59, V_HTyr100	0.10%
44.	1rjl	4	V_LTrp32, V_HTrp33, V_HTyr101, V_HTyr105	0.09%
45.	1sy6	6	V_LTyr31, V_LTrp90, V_HTyr50, V_HTyr57, V_HTyr99, V_HTyr104	0.05%
46.	1tpx	4	/	/
47.	1tzh	7	V_HTyr50, V_HTyr56, V_HTyr100B	26.09%
48.	1tzi	5	V_LTyr29, V_LTyr92	0.00%
49.	1uac	6	V_LTyr50, V_LTrp94, V_LTyr96, V_HTyr33, V_HTyr50, V_HTrp98	0.01%
50.	1uj3	5	V_LTyr50, V_LTyr96, V_HTyr332, V_HTyr333, V_HTyr402	0.09%
51.	1v7m	7	V_LTyr31, V_LTyr33, V_LTyr48, V_LTyr93, V_HTrp33	3.69%
52.	1w72	5	V_LTrp91, V_HTyr100A, V_HTyr100B, V_HTyr100C	0.55%
53.	1wej	4	V_LTyr32, V_LTrp92, V_HTyr33, V_HTyr101	1.53%
54.	1xf5	5	V_LTyr38, V_LTrp56, V_LTyr98, V_HTrp47, V_HTrp50	0.15%

55.	1xiw	6	V_LTyr33, V_LTrp97, V_HTyr54, V_HTyr101, V_HTyr102, V_HTrp107	0.08%
56.	1ymh	0	/	/
57.	1yjd	7	V_LTyr30, V_LTrp32, V_HTyr33, V_HTyr52, V_HTyr101 V_HTrp105,	0.61%
58.	1yqv	7	V_LTyr32, V_LTyr34, V_LTrp91, V_HTrp47	8.59%
59.	1yy9	6	V_LTrp94, V_HTrp52, V_HTyr101, V_HTyr102, V_HTyr104	0.58%
60.	1ztx	6	V_LTrp50, , V_HTyr27, V_HTyr32, V_HTyr98	8.99%
61.	2aep	5	V_LTyr49, V_LTyr53, V_LTyr94, V_HTyr33	1.71%
62.	2arj	7	V_LTyr49, V_LTyr50, V_LTyr53, V_LTyr92, V_HTrp52, V_HTrp99, V_HTyr100	0.02%
63.	2b2x	6	V_LTyr48, V_LTyr52, V_LTrp90, V_LTrp95, V_HTrp47, V_HTyr58	0.00%
64.	2bob	4	/	/
65.	2dd8	3	V_LTrp91, V_LTyr96	/
66.	2eiz	9	V_LTyr49, V_LTyr50, V_LTrp94, V_LTyr96, V_HTyr33, V_HTyr50, V_LTyr53, V_HTyr58, V_HTrp98	0.00%
67.	2fd6	7	V_LTyr32, V_LTrp91, V_LTyr93, V_HTyr33, V_HTrp50, V_HTrp95, V_HTrp99	0.10%
68.	2fjg	4	V_LTyr92, V_HTyr105	65.08%
69.	2ghw	0	/	/

70.	2i5y	2	/	/
71.	2j4w	8	V_LTyr32, V_LTrp91, V_HTyr32, V_HTyr33, V_HTyr35, V_HTyr56, V_HTyr58, V_HTyr100C	0.01%
72.	2j5l	8	V_LTyr32, V_LTrp91, V_HTyr32, V_HTyr33, V_HTyr35, V_HTyr56, V_HTyr58, V_HTyr100C	0.00%
73.	2j6e	6	V_LTyr32, V_LTyr34, V_LTrp91, V_HTyr35, V_HTyr58, V_HTyr100	0.11%
74.	2jel	5	V_LTyr32, V_LTyr96, V_HTyr100	13.19%
75.	2j88	9	V_LTyr30, V_LTyr32, V_LTyr36, V_LTyr92, V_HTyr52, V_HTyr53, V_HTyr95	0.24%
76.	2nr6	6	V_LTyr49, V_LTyr53	66.18%
77.	2q8a	3	V_LTyr49, V_LTyr50, V_LTyr55	/
78.	2qqk	6	V_LTyr94, V_HTyr52, V_HTyr56 , V_HTrp100D, V_HTyr100E	3.14%
79.	2qqn	4	V_LTyr32, V_HTyr99, V_HTyr100	42.04%
80.	2uzi	3	V_HTyr50, V_HTyr59	/
81.	2zch	7	V_HTyr32, V_HTyr33, V_HTyr58, V_HTyr97	3.82%
82.	3bt2	7	V_LTyr32, V_LTrp91, V_LTyr93, V_HTyr33, V_HTrp50, V_HTrp95, V_HTrp99	0.20%
