# Supplementary material for: Stacking and energetic contribution of aromatic islands at the binding interface of antibody proteins
Source: Immunome Res. 2010 Sep 27;6(Suppl 1):S1. doi: 10.1186/1745-7580-6-S1-S1 (PMC2946779; doi:10.1186/1745-7580-6-S1-S1)
Supplement: Additional File 3 — Table S3. Experimental Binding affinity for immune complexes [file 1745-7580-6-S1-S1-S3.pdf]

Additional File 3

Table S3. Experimental Binding affinity for immune complexes

PDB ID	Antibody	Antigen	Binding Affinity	Condition	Reference
1a2y	IgG1-kappa D1.3 Fv	lysozyme	k_{on} = (8.0+/-2.4)×10^7 $M^{-1}s^{-1}$	25°C	1
1ahw	immunoglobulin Fab 5G9	tissue factor	k_{on} = (3.4+/-0.3)×10^9 $M^{-1}s^{-1}$<br>△G = -11.5 kcal/mol	25°C	2
1bj1	vascular endothelial growth factor	Fab fragment of a humanized neutralizing antibody	k_{on} = 4.1×10^4 $M^{-1}s^{-1}$<br>k_{off} = 1.4×10^{-4} s^{-1}<br>K_D = 3.4+/-0.9 nM	25°C	3
1dqj	Fab of anti-hen egg white lysozyme antibody HyHEL-63	hen egg white lysozyme	K_A = 3.5×10^8 M^{-1}	25°C	4
1e6j	Fab 13B5	RH24(a 243 residue recombinant protein from HIV-1 capsid protein p24)	k_{on} = 3.5×10^5 $M^{-1}s^{-1}$<br>k_{off} = 1.2×10^{-3} s^{-1}<br>K_D = 29nM	25°C	5
1eo8	Fab fragment of antibody BH151	hemagglutinin of influenza virus	K_D = 8.9±2 nM	22°C	6
1fbi	IgG1 F9.13.7 Fab	guinea fowl lysozyme	K_A = 10^8 M^{-1}	25°C	7

1h0d	the Fab fragment of 26-2F	human angiogenin	K_D = 1.6nM	25°C	8
1jhl	IgG1-kappa D11.15 FV	pheasant egg lysozyme	$k_{on} = 3.3\times10^{-6}$ $M^{-1}s^{-1}$<br>$k_{off} = 2.2\times10^{4}$ s^{-1}<br>$K_A = 15\times10^{-9}$ M^{-1}	25°C	9
1jps	humanized- ab fragment D3h44	tissue factor	$K_D = 0.1\times10^{-9}$ M	25°C	10
1jrh	A6 Fab	interferon-gamma receptor a-chain	K_D = 14.2 nM	25°C	11
1ndg	H8	hen egg white lysozyme	$K_A = 5.0(+/-0.2)\times10^{9}$ M^{-1}	25°C	12
1ndm	H26	hen egg white lysozyme	$K_A = 1.4(+/-0.1)\times10^{8}$ M^{-1}	25°C	12
1nsn	antibody N10 Fab fragment	staphylococcal nuclease	$K_A = 4.4\times10^{8}$ M^{-1}<br>$\triangle G$ = -12 kcal/mol	25°C	13
1oaz	antibody SPE7, a monoclonal immunoglobulin E (IgE)	trx-Shear3	K_D = ~10 mM	25°C	14
1ob1	the Fab fragment of the monoclonal antibody G17.12	merozoite surface protein 1	$K_D = 2.0\times10^{-10}$ M	25°C	15
1qfu	HC45 Fab	influenza hemagglutinin	K_D =3+/-1nM	22°C	16

1sy6	mAb OKT3 Fab	T cell receptor CD3-gamma/epsilon	k_{on} = 19.7+/-5.93×10^{-4} s^{-1}M^{-1}<br>k_{off} = 0.48 s^{-1}<br>K_D = 2.63 mM	25°C	17
1tzh	Fab YADS1	human vascular endothelial growth factor (hVEGF)	k_{on} = 3×10^{5} s^{-1}M^{-1}<br>k_{off} = 5×10^{-4} s^{-1}<br>K_D = 1.8+/-0.3 nM	25°C	18
1tzi	Fab YADS2	human vascular endothelial growth factor (hVEGF)	k_{on} = 1×106 s^{-1}M^{-1}<br>k_{off} = 1×10^{-2} s^{-1}<br>K_D = 10 +/- 2 nM	25°C	18
1v7m	TN1 Fab	thrombopoietin	K_A ≈ 10^{9} M^{-1}	25°C	19
1yy9	cetuximab Fab C225	epidermal growth factor receptor	K_D = 2.3 ± 0.5 nM	25°C	20
2aep	Fab of monoclonal antibody Mem5	Neuraminidase (NA) of influenza virus	K_D = 10.9 ± 3.0 nM	25°C	28
2b2x	antibody AQC2 Fab	integrin alpha-1	k_{on} = 2.5×10^{-5} s^{-1}<br>k_{off} = 2.6×10^{-3} M^{-1}s^{-1}<br>K_D = 10.7 nM	25°C	21

2dd8	Fab m396	spike glycoprotein	k_{on} = 3.0+/-0.3×10^{5} M^{-1}s^{-1}<br>k_{off} = 6.1+/-0.6×10^{-3} s^{-1}<br>K_D = 20 nM	25°C	22
2fjg	G6 Fab	vascular endothelial growth factor A	K_D = 1.5 nM	25°C	23
2j4w	Fab fragment of monoclonal antibody F8.12.19	P. vivax apical membrane antigen 1	k_{on} = 1.6×10^{5} M^{-1}s^{-1}<br>k_{off} = 1.0×10^{-4} s^{-1}<br>K_D = 0.6 nM	25°C	24
2j5l	Fab fragment of monoclonal antibody F8.12.19	P. falciparum apical membrane antigen 1	k_{on} = 2.2×10^{4} M^{-1}s^{-1}<br>k_{off} = 1.7×10^{-3} s^{-1}<br>K_D = 77 nM	25°C	24
2jel	JEL42 Fab	histidine-containing protein	K_D = 3.7+/-0.3 nM	23°C	25
2qqn	anti-panNrp^{A}	neuropilin-1	0.21nM	25°C	26
2uzi	anti-RAS Fv	GTPase HRAS	k_{on} = 3.37×10^{6} M^{-1}s^{-1}<br>k_{off} = 1.17×10^{-3} s^{-1}<br>K_D = 0.35 nM	25°C	27

References

1. Dall'Acqua W, Goldman ER, Lin WH, Teng C, Tsuchiya D, Li HM, Ysern X, Braden BC, Li YL, Smith-Gill SJ, Mariuzza RA: **A mutational analysis of binding interactions in an antigen-antibody protein-protein complex**. *Biochemistry* 1998, **37**:7981 -7991.
2. Huang M, Syed R, Stura EA, Stone MJ, Stefanko RS, Ruf W, Edgington TS, Rees IDAC. **The mechanism of an inhibitory antibody on TF-initiated blood coagulation revealed by the crystal structures of human tissue factor, Fab 5G9 and TFG9 complex**. *J. Mol. Biol.* 1998, **275**:873-894.
3. Muller YA, Chen Y, Christinger HW, Li B, Cunningham BC, Lowman HB, de Vos AM. **VEGF and the Fab fragment of a humanized neutralizing antibody: crystal structure of the complex at 2.4Å resolution and mutational analysis of the interface**. *Structure* 1998, **6**:1153-1167.
4. Li YL, Li HM, Smith-Gill SJ, Mariuzza RA. **Three-dimensional structures of the free and antigen-bound fab from monoclonal antilysozyme antibody HyHEL-63**. *Biochemistry* 2000,**39**:6296-6309.
5. Monaco-Malbet S, Berther-Colominas C, Novelli A, Battai N, Piga N, Cheynet V, Mallet F, Cusack S. **Mutual conformational adaptations in antigen and antibody upon complex formation between an Fab and HIV-1 capsid protein p24**. *Structure* 2000,**8**:1069-1077.
6. Fleury D, Daniels RS, Skehel JJ, Knossow M, Bizebard T. **Structural evidence for recognition of a single epitope by two distinct antibodies**. *Proteins* 2000,**40**:572-578.
7. Lescar J, Pellegrini M, Souchon, H, Tello D, Poljak RJ, Peterson N, Greene M, Alzari PM. **Crystal Structure of a Cross-reaction Complex between Fab F9.13.7 and Guinea Fowl Lysozyme** *J. Biol. Chem* 1995,**270**:18067-18076.
8. Chavali G, Papageorgiou A, Olson K, Fett J, Hu G, Shapiro R, Acharya K. **The crystal structure of human angiogenin in complex with an antitumor neutralizing antibody**. *Structure* 2003,**11**:875-885.
9. Chitarra V, Alzari PM, Bentley GA, Bhat TN, Eiselé JL, Houdusse A, Lescar J, Souchon H, Poljak RJ. **Three-dimensional structure of a heteroclitic antigen-antibody cross-reaction complex**. *Proc. Natl. Acad. Sci.* 1993,**90**:7711-7715.
10. Faelber K, Kirchhofer D, Presta L, Kelley RF, Muller YA. **The 1.85 Å resolution crystal structures of tissue factor in complex with humanized fab d3h44 and of free humanized fab d3h44: revisiting the solvation of antigen combining sites**. *J Mol. Biol.* 2001,**313**:83-97.
11. Sogabe S, Stuart F, Henke C, Bridges A, Williams G, Birch A, Winkler FK, Robinson JA. **Neutralizing epitopes on the extracellular interferon gamma receptor (IFN-gamma-R) a-chain characterized by homolog scanning mutagenesis and X-ray crystal structure of the A6 Fab-IFN-gamma-R1-108 complex.** *J Mol. Biol*. 1997,**273**:882-897.
12. Li YL, Li HM, Yang F, Smith-Gill SJ, Mariuzza RA. **X-ray snapshots of the maturation of an antibody response to a protein antigen.** *Nature Structural Biology* 2003,**10**:482-488.
13. Bossart-Whitaker P, Chang CY, Novotny J, Benjamin DC, Sheriff S. **The crystal structure of the antibody N10–staphylococcal nuclease complex at 2.9 Å resolution.** *J. Mol. Biol.* 1995,**253**:559-575.
14. James LC, Roversi P, Tawfik DS. **Antibody multispecificity mediated by conformational diversity.** *Science* 2003,**299**:1362-1367.
15. Pizarro JC, Chitarra V, Verger D, Holm I, Pêtres S, Dartevelle S, Nato F, Longacre S, Bentley GA. **Crystal structure of a Fab complex formed with PfMSP1-19, the C-terminal fragment of merozoite surface protein 1 from plasmodium falciparum: a malaria vaccine candidate.** *J. Mol. Biol.* 2003,**328**:1091-1103.
16. Fleury D, Barrère B, Bizebard T, Daniels RS, Skehel JJ, Knossow M. **A complex of influenza hemagglutinin with a neutralizing antibody that binds outside the virus receptor binding site.** *Nature Struct. Biol.* 1999,**6**:530-534.

17. Kjer-Nielsen L, Dunstone MA, Kostenko L, Ely LK, Beddoe T, Mifsud NA, Purcell AW, Brooks AG, McCluskey J, Rossjohn J. **Crystal structure of the human T cell receptor CD3-gamma/epsilon heterodimer complexed to the therapeutic mAb OKT3**. *Proc. Natl. Acad. Sci.* 2004,**101**:7675-7680.
18. Fellouse FA, Wiesmann C, Sidhu SS. **Synthetic antibodies from a four-amino-acid code: A dominant role for tyrosine in antigen recognition**. *Proc. Natl. Acad. Sci.* 2004,**101**:12467-12472.
19. Feese MD, Tamada T, Kato Y, Maeda Y, Hirose M, Matsukura Y, Shigematsu H, Muto T, Matsumoto A, Watarai H, Ogami K, Tahara T, Kato T, Miyazaki H, Kuroki R. **Structure of the receptor-binding domain of human thrombopoietin determined by complexation with a neutralizing antibody fragment.** *Proc. Natl. Acad. Sci.* 2004,**101**:1816-1821.
20. Li S, Schmitz K, Jeffrey P, Wiltzius J, Kussie P, Ferguson K. **Structural basis for inhibition of the epidermal growth factor receptor by cetuximab**. *Cancer Cell* 2005,**7**:901-311.
21. Clark LA, Boriack-Sjodin PA, Eldredge J, Fitch C, Friedman B, Hanf KJM, Jarpe M, Liparoto SF, Li Y, Lugovskoy A, Miller S, Rushe M, Sherman W, Simon K, Van Vlijman H. **Affinity enhancement of an in vivo matured therapeutic antibody using structure-based computational design.** *Protein Sci.* 2006,**15**:949-960.
22. Prakakaran P, Gan JH, Feng Y, Zhu ZY, Choudhry V, Xiao XD, Ji XH, Dimitrov DS. **Structure of Severe Acute Respiratory Syndrome Coronavirus Receptor-binding Domain Complexed with Neutralizing Antibody**. *J. Mol. Biol.* 2006,**281**:15829-15836.
23. Fuh G, Wu P, Liang WC, Ultsch M, Lee CV, Moffat B, Wiesmann C. **Structure-Function Studies of Two Synthetic Anti-vascular Endothelial Growth Factor Fabs and Comparison with the AvastinTM Fab**. *J. Mol. Chem.* 2006,**281**:6625-6631.
24. Igonet S, Normand BVL, Faure G, Riottot MM, Kocken CHM, Thomas AW, Bentley GA. **Cross-reactivity studies of an anti-plasmodium vivax Apical membrane antigen 1 monoclonal antibody: binding and structural characterization**. *J. Mol. Biol.* 2007,**366**:1523-1537.
25. Prasad L, Waygood EB, Lee JS, Delbare LTJ. **The 2.5 A resolution structure of the jel42 Fab fragment/HPr complex**. *J. Mol. Biol.* 1998,**280**:829-845.
26. Appleton BA, Wu P, Maloney J, Yin JP, Liang WC, Stawicki S, Mortara K, Bowman KK, Elliott, JM, Desmarais W, Bazan JF, Bagri A, Tessier-Lavigne M, Koch AW, Wu Y, Watts RJ, Wiesmann C. **Structural studies of neuropilin/antibody complexes provide insights into semaphorin and VEGF binding**. *EMBO J.* 2007,**26**:4902-4912.
27. Tanaka T, Williams RL, Rabbitts TH. **Tumour prevention by a single antibody domain targeting the interaction of signal transduction proteins with ras**. *EMBO J.* 2007,**26**:3250-3259.
