# Supplementary material for: Stacking and energetic contribution of aromatic islands at the binding interface of antibody proteins
Source: Immunome Res. 2010 Sep 27;6(Suppl 1):S1. doi: 10.1186/1745-7580-6-S1-S1 (PMC2946779; doi:10.1186/1745-7580-6-S1-S1)
Supplement: Additional file 4 — Figure S1 Correlation between energetic gap and SASA loss of Aromatic Islands at 30 antibody interfaces. [file 1745-7580-6-S1-S1-S4.pdf]

Additional file 4

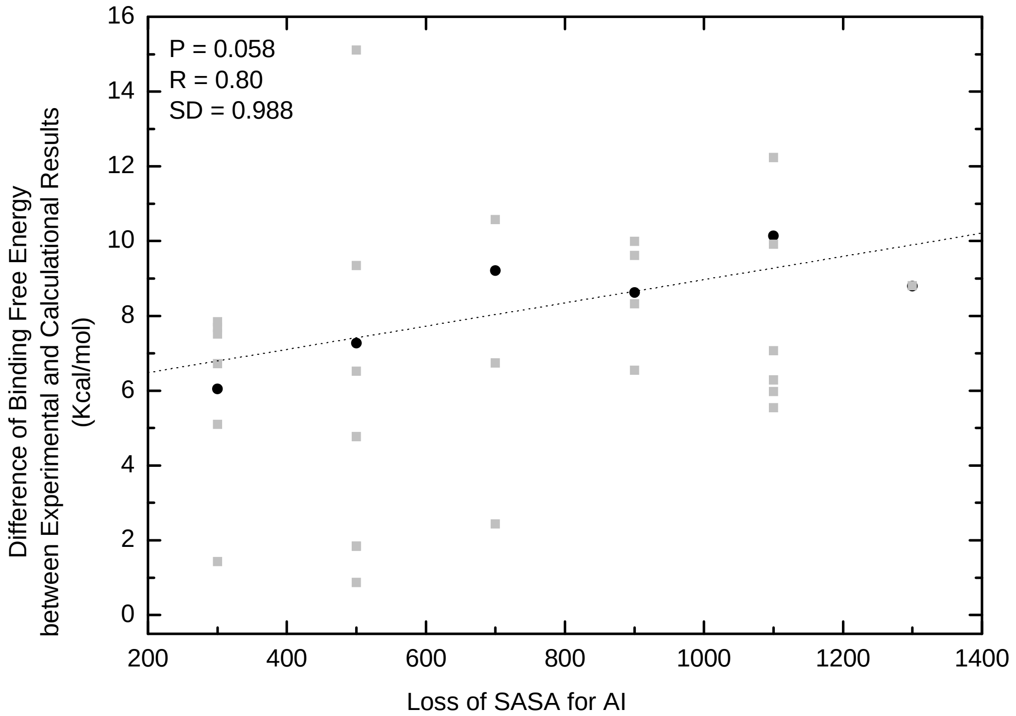

Figure S1 Correlation between energetic gap and SASA loss of Aromatic Islands at 30 antibody interfaces.

The computational binding free energy is calculated with Rosetta method. Along the x-axis all of the values in a bin (size 200 Å^2) are pulled together as a group and shown in the middle. The gap of binding free energy between theoretical and experimental data is indicated with grey square for every immune complex. In each group, the gap is averaged and indicated with black dot.
